# Product and Equipment Coding in National Practitioner Data Bank Malpractice Payment Reports, 2019-2025

**DOI:** 10.7759/cureus.115781

**Published:** 2026-09-04

**Authors:** Tucker Bettis, Quynh Tran, Logan Glosser, Felipe Maegawa, Snehal Patel

**Affiliations:** 1 General Surgery, Emory University Hospital, Atlanta, USA

**Keywords:** health services research, medical devices, medical malpractice, national practitioner data bank, patient safety

## Abstract

Introduction: Codes related to products and equipment in the National Practitioner Data Bank (NPDB) may identify only a selected subset of malpractice payment reports involving medical products. Our objective was to compare coded injury severity and payment profiles between physician malpractice payment reports with and without product or equipment coding, and to determine whether any differences reflected allegation-group composition, so that researchers can judge whether these codes are usable as a standalone case definition for product-related malpractice.

Methods: We conducted a retrospective cross-sectional analysis of United States physician malpractice payment reports in the NPDB Public Use File with report origination years from 2019 through 2025, the seven most recent complete origination years in the file. Product or equipment coding was identified from the allegation group and allegation code fields. Payments are released in the Public Use File as categorical bands representing the payment made by the payer for the named practitioner; each band was represented by its midpoint. We compared coded injury severity and banded payment distributions and estimated associations with severe injury or death before and after adjustment for allegation group.

Results: Among 54,351 reports, 589 (1.08%) had product or equipment coding. Severe injury or death was coded in 77 product/equipment-coded reports (13.1%) and 26,467 non-product/equipment-coded reports (49.2%); death was coded in 40 product/equipment-coded reports (6.8%) and 17,240 non-product/equipment-coded reports (32.1%). Median payment-band midpoint values were $97,500 for product/equipment-coded reports and $265,000 for non-product/equipment-coded reports. The association between product or equipment coding and lower odds of coded severe injury or death persisted after adjustment for allegation group (adjusted odds ratio, 0.29; 95% confidence interval, 0.21-0.40).

Conclusions: Physician malpractice payment reports containing product or equipment codes were rare and had lower coded injury severity and payment values than other physician reports. These findings describe how claims are coded rather than how severely patients were injured, and the codes should not be used alone to define or estimate the malpractice burden associated with medical products.

## Introduction

The National Practitioner Data Bank (NPDB) is a widely used source for studying malpractice payments and practitioner accountability in the United States [1-3]. Health services researchers use its publicly available fields to describe the content, severity, and payment profiles of malpractice reports. The interpretation of these fields, however, depends on how claims are coded.

Product and equipment codes provide a useful test of this measurement problem. Modern procedural care routinely involves implants, instruments, supplies, monitoring equipment, surgical energy devices, robotic platforms, and other medical products. Injuries involving products may nevertheless be coded according to the clinical act that produced liability rather than a product or equipment field. Medical device adverse-event reporting also occurs in United States Food and Drug Administration systems such as the Manufacturer and User Facility Device Experience database, but those systems are not malpractice payment databases and cannot establish incidence or causation [4,5].

The objectives of this study were threefold: to develop a reproducible, field-level classification of NPDB physician malpractice payment reports with product or equipment coding; to compare coded injury severity and payment profiles between reports with and without such coding; and to determine whether any observed difference reflected the composition of allegation groups and persisted in sensitivity analyses restricted to single-practitioner claims and earlier report years. The applied purpose is practical rather than clinical: investigators, policy analysts, and medicolegal researchers increasingly use NPDB allegation fields to characterize device- and product-associated liability, and this study asks whether those fields can carry that weight on their own. We did not attempt to validate the codes against claim records or to estimate the incidence of product-related patient harm.

## Materials and methods

Study design and data source

We conducted a retrospective cross-sectional analysis of the NPDB Public Use File containing reports processed through March 31, 2026 [1]. The study population included malpractice payment reports for United States physicians with Doctor of Medicine (MD) or Doctor of Osteopathic Medicine (DO) licenses (LICNFELD codes 10 and 20) and report origination years (ORIGYEAR) from 2019 through 2025. ORIGYEAR was selected because the analysis evaluates the reporting system's output rather than the timing of the underlying alleged act.

The study period was defined by data completeness. The 2019 through 2025 window comprises the seven most recent complete report-origination years contained in the file and reflects contemporary allegation-coding practice. Reports with an origination year of 2026 were excluded because the file captured only the first quarter of that year; a partial year would have contributed an incomplete denominator, and because malpractice payment reports are submitted after payment with a variable administrative lag, recent months are also incompletely populated relative to earlier years. Origination years before 2019 were excluded to keep the cohort within a single recent coding era; a sensitivity analysis restricted to ORIGYEAR 2019-2023 tested whether the findings depended on the most recent report years. All reports meeting the license-field and origination-year criteria were included, with no exclusions based on allegation, outcome, or payment values.

The unit of analysis was the individual NPDB report. Reports that may have arisen from the same underlying incident were not combined because the Public Use File is organized at the report level and the study evaluates information visible in report-level coding.

Data processing and missing data

Field values were used as released in the Public Use File. Classification variables were derived directly from the published field codes, and no records were merged, de-duplicated, recoded, or otherwise modified beyond the definitions specified below. No imputation was performed. Analyses were conducted on complete cases with respect to the fields required for each analysis; a report with a missing or unknown value in a required field did not contribute to that specific analysis but remained eligible for others. Analytic code is available from the corresponding author upon reasonable request.

Exposure classification

A report was classified as having product or equipment coding when the allegation group (ALGNNATR) equaled 80 (Equipment/Product Related) or either allegation field (ALEGATN1 or ALEGATN2) contained code 303 (equipment utilization problem), 320 (problem with appliance, prosthesis, orthotic, or device), 332 (wrong equipment), 704 (equipment malfunction), 709 (failure to test equipment), or 715 (product liability). We also described a narrower product-intrinsic subset defined by codes 320, 704, or 715, and an explicit product-liability subset defined by code 715. The complete field-and-code specification is provided in the Appendix.

These components are not independent of the allegation-group variable used for adjustment. A report assigned to allegation group 80 is classified as exposed by definition, so an allegation-group-adjusted model necessarily contrasts coded and uncoded reports within clinical allegation groups rather than across the full cohort. This structural overlap is carried forward into the interpretation of the adjusted estimate and is addressed in the Discussion.

Payment variable and midpoint derivation

The NPDB Public Use File documentation defines TOTALPMT as the total payment made by the payer for the practitioner named in the report and releases it as a categorical band rather than an exact dollar value [1]. TOTALPMT is therefore a banded practitioner-level payment, not a verified claim-level indemnity total, and reports arising from a single underlying claim involving several practitioners may each carry a separate banded value.

Each report was assigned the arithmetic midpoint of the lower and upper bounds of its published band. The highest band is open-ended and was assigned its lower bound, a conservative convention that cannot inflate the values reported for either group. All payment figures reported here, including medians and interquartile ranges, are therefore band-derived approximations and should not be read as exact payments.

Outcomes and statistical analysis

The primary outcomes were coded as severe injury or death and banded payment. Severe injury or death comprised the NPDB OUTCOME categories for significant permanent injury, major permanent injury, quadriplegia, brain damage or lifelong care, and death. Death was also examined separately.

We calculated report counts, proportions, Wilson 95% confidence intervals, and medians with interquartile ranges. Payment distributions were compared using the Mann-Whitney U test, and severity proportions were compared using uncorrected chi-square tests. Because midpoint assignment is a strictly monotonic transformation of the underlying bands, the rank-based Mann-Whitney comparison is equivalent to comparing the ordered payment-band distributions and does not depend on the particular dollar value assigned within a band. The transformation does, however, assign identical values to all reports within a band, which creates extensive ties, removes within-band variation, and makes the reported medians and interquartile ranges band-level approximations. Small differences in payment distributions should not be overinterpreted on this basis; the comparison supports a statement about ordered payment bands rather than about dollar amounts.

Logistic regression was used to estimate crude and adjusted associations between product or equipment coding and coded severe injury or death, with reports lacking product or equipment coding as the reference category. Adjusted models added allegation group (ALGNNATR) as a set of categorical indicator variables with a single omitted reference group; no interaction terms were fitted, and no variable selection was performed. Odds ratios are reported with Wald 95% confidence intervals. Because the same underlying claim may generate several practitioner reports, we repeated the severity and payment analyses among claims involving a single practitioner (NUMBPRSN=1). Subgroup comparisons within allegation groups were limited in advance to the surgery-related and treatment-related groups, the two clinical groups containing enough product- or equipment-coded reports for a stable comparison. Analyses were performed in Python version 3.12 (Python Software Foundation, Fredericksburg, VA, USA) using pandas, NumPy, SciPy, and statsmodels.

Ethical considerations

This study used deidentified, publicly available data from the NPDB Public Use File and did not involve identifiable private information. It did not constitute human subjects research, and institutional review board review was not required.

## Results

Prevalence of product or equipment coding

From 2019 through 2025, the NPDB contained 54,351 malpractice payment reports for United States MD and DO physicians. Product or equipment coding appeared in 589 reports (1.08%; 95% confidence interval, 1.00%-1.17%). A narrower product-intrinsic subset appeared in 194 reports (0.36%), and explicit product-liability coding appeared in 11 reports (0.02%) (Table 1).

**Table 1:** Product and equipment coding definitions and prevalence in physician malpractice payment reports, 2019-2025 (n=54,351)

Classification	Definition	Reports	% of all reports
Product or equipment coding	Allegation group 80 or either allegation field with code 303, 320, 332, 704, 709, or 715	589	1.08
Intrinsic product subset	Either allegation field with code 320, 704, or 715	194	0.36
Explicit product liability	Either allegation field with code 715	11	0.02

Severity and payment profiles

Severe injury or death was coded in 77 of 589 reports with product or equipment coding (13.1%) and 26,467 of 53,762 other reports (49.2%), a difference of 36.2 percentage points. Death was coded in 40 of 589 reports with product or equipment coding (6.8%) and 17,240 of 53,762 other reports (32.1%), a difference of 25.3 percentage points. Median payment-band midpoint values were also lower among reports with product or equipment coding: $97,500 (interquartile range, $32,500-$285,000) versus $265,000 (interquartile range, $97,500-$545,000) (Table 2). The corresponding uncorrected chi-square and Mann-Whitney U test results are shown in Table 3.

**Table 2:** Characteristics of physician malpractice payment reports by product or equipment coding status, 2019-2025 (n=54,351) IQR, interquartile range; NPDB, National Practitioner Data Bank; TOTALPMT, NPDB Public Use File banded payment made by the payer for the named practitioner, represented here by band midpoints rather than exact payment amounts

Coding status	Reports, n (%)	TOTALPMT midpoint, median (IQR)	Death, n (%)	Severe injury or death, n (%)
Product or equipment coding	589 (1.08)	$97,500 ($32,500-$285,000)	40 (6.8)	77 (13.1)
No product or equipment coding	53,762 (98.92)	$265,000 ($97,500-$545,000)	17,240 (32.1)	26,467 (49.2)

**Table 3:** Statistical comparisons by product or equipment coding status, 2019-2025 (n=54,351) The Mann-Whitney U test compared the full distributions of payment-band midpoints, not only the displayed medians; because midpoint assignment preserves band order, this comparison is equivalent to a comparison of ordered payment bands. TOTALPMT, NPDB Public Use File banded payment made by the payer for the named practitioner; NPDB, National Practitioner Data Bank

Comparison	Product/equipment-coded	Other reports	Test	Statistic	P value
Severe injury or death	77/589 (13.1%)	26,467/53,762 (49.2%)	Uncorrected chi-square	304.832	<0.001
Death	40/589 (6.8%)	17,240/53,762 (32.1%)	Uncorrected chi-square	171.648	<0.001
TOTALPMT midpoint distribution	$97,500 median	$265,000 median	Mann-Whitney U	10,338,784	<0.001
Severe injury/death within coded reports	Equipment/Product group: 34/271 (12.5%)	Clinical allegation groups: 43/318 (13.5%)	Uncorrected chi-square	0.117	0.73

Allegation group and sensitivity analyses

Reports with product or equipment coding were concentrated in particular allegation groups. Of the 589 coded reports, 318 (54.0%) were assigned to a clinical allegation group, including 243 (41.3%) related to surgery, rather than the Equipment/Product group. Within the coded subset, severe injury or death was coded in 34 of 271 Equipment/Product group reports (12.5%) and 43 of 318 clinical allegation group reports (13.5%; P=0.73).

The severity gradient also persisted within the two clinical allegation groups examined. Surgical reports with product or equipment coding had severe injury or death in 31 of 243 reports (12.8%) compared with 3,926 of 15,119 other surgical reports (26.0%), as did treatment reports with product or equipment coding compared with other treatment reports (5 of 46 (10.9%) versus 6,392 of 11,332 (56.4%)). Surgery-related and treatment-related reports accounted for 289 of the 318 coded reports assigned to clinical allegation groups; the remaining 29 coded reports were dispersed across the other clinical groups in numbers too small to support stable subgroup estimates, and no further allegation-group subgroups were analyzed. Product or equipment coding remained associated with lower odds of coded severe injury or death after adjustment for allegation group (odds ratio, 0.29; 95% confidence interval, 0.21-0.40), compared with a crude odds ratio of 0.16 (95% confidence interval, 0.12-0.20) (Table 4).

**Table 4:** Logistic regression associations between product or equipment coding and severe injury or death Odds ratios compare reports with product or equipment coding with all other reports (reference category). Adjusted models included ALGNNATR as categorical indicator variables. Because allegation group 80 also forms part of the exposure definition, adjusted estimates contrast coded and uncoded reports within clinical allegation groups and describe differences in coded severity rather than an effect of product or equipment involvement on injury. TOTALPMT values are midpoints of NPDB public use payment bands and represent the payment made by the payer for the named practitioner rather than exact practitioner-level payments. ALGNNATR, allegation group; TOTALPMT, NPDB Public Use File banded payment made by the payer for the named practitioner; NPDB, National Practitioner Data Bank

Model	Analytic cohort	Odds ratio	95% confidence interval	P value
Crude	Full cohort (n=54,351)	0.16	0.12-0.20	<0.001
Adjusted for allegation group	Full cohort (n=54,351)	0.29	0.21-0.40	<0.001
Single-practitioner sensitivity	NUMBPRSN=1 (n=43,130)	0.32	0.22-0.45	<0.001
Report-year sensitivity	2019-2023 cohort (n=37,854)	0.32	0.22-0.46	<0.001

The severity gradient was stable in sensitivity analyses. Among claims involving one practitioner, severe injury or death was coded in 66 of 520 reports with product or equipment coding (12.7%) and 19,454 of 42,610 other reports (45.7%). Corresponding payment-band midpoint medians were $101,250 (interquartile range, $36,250-$285,000) and $275,000 (interquartile range, $105,000-$565,000), and the adjusted odds ratio was 0.32 (95% confidence interval, 0.22-0.45). In the 2019-2023 window, severe injury or death was coded in 55 of 428 reports with product or equipment coding (12.9%) and 18,321 of 37,426 other reports (49.0%), with an adjusted odds ratio of 0.32 (95% confidence interval, 0.22-0.46).

## Discussion

The central finding of this analysis is a measurement finding. Product and equipment codes in the NPDB mark a small and atypical slice of physician malpractice payment reports rather than a representative sample of paid claims involving medical products, and the direction of that selection matters for anyone using the fields as a case definition. Taken at face value, the lower coded severity and lower banded payments observed among coded reports would suggest that product-related malpractice is comparatively benign. Read as a property of the coding system, which is what a cross-sectional analysis of report-level fields can support, the same pattern indicates that a researcher who equates these codes with product-related harm will understate both how often such harm reaches a paid claim and how severe it is when it does. The practical implication is that these fields should be treated as indicators of what was documented, not as measures of what occurred.

Several mechanisms could produce this selection, none of which the present design can test. Allegation coding may follow the clinical act alleged to be negligent, so that a catastrophic outcome involving an implant or an energy device is recorded as a surgical or treatment allegation and the product dimension is not captured in a separate field. Claims in which a device is central may also involve the manufacturer as a party, and compensation paid solely by a manufacturer falls outside the NPDB entirely, so the practitioner-attributed payment that reaches the file may represent only part of a larger resolution. It is also possible that the events most naturally described by codes such as equipment malfunction, wrong equipment, or failure to test equipment are disproportionately those producing moderate or recoverable injury. These explanations are speculative and are offered to direct future validation work rather than as findings.

Prior national paid-claims studies have used the NPDB to describe physician malpractice patterns by specialty and claim concentration [2,3]. Our findings add a narrower measurement point at the level of the allegation fields themselves.

The allegation group analysis helps locate the phenomenon. Reports with product or equipment coding were distributed between the Equipment/Product group and clinical allegation groups, particularly Surgery Related, and coded severity was similar in both, so the low-severity profile is not simply an artifact of which group these reports landed in.

The adjusted odds ratio requires careful reading because of the overlap described in the Methods. Allegation group 80 is one component of the exposure definition, so adjusting for allegation group does not remove confounding in the usual sense; it restricts the informative contrast largely to coded and uncoded reports within clinical allegation groups. The adjusted estimate therefore describes a difference in how severity is coded between otherwise similarly classified reports. It is not an estimate of an effect of product or equipment involvement on patient injury, and nothing in these data indicates that the presence of a device makes an injury less severe.

These patterns differ from device-event surveillance systems such as the FDA Manufacturer and User Facility Device Experience database, which is designed for adverse-event reporting rather than malpractice payment reporting and cannot be used to estimate incidence or causation [4,5]. The NPDB has the opposite limitation for this question: it records paid malpractice reports but lacks device names, product identifiers, narratives, and manufacturer-only compensation. The low-severity profile also contrasts with literature on retained foreign objects and surgical never events, where malpractice-claim and hospital-based studies have emphasized preventable harm, systems factors, and clinically meaningful morbidity, and with court-record studies of robot-assisted surgery malpractice, in which allegations often combine negligent surgery, training, informed consent, and product-adjacent issues rather than isolating a single device-causation pathway [6-11]. That contrast is itself informative: the harms described in those literatures are largely invisible in the NPDB product and equipment fields. Studies evaluating compensation for product-associated injuries will require complementary data sources, record linkage, or validation against claim narratives.

Limitations

This study has several limitations. First, it was descriptive and cross-sectional at the report level; the associations reported here cannot support causal interpretation, and they describe coded documentation rather than patient outcomes. Second, we did not validate product or equipment codes against a gold standard, so sensitivity, specificity, and coding accuracy are unknown. Exposure misclassification is likely and may well be differential: if the product dimension is more often omitted for catastrophic events that are coded to the clinical act instead, part or all of the observed severity gradient would be an artifact of that omission rather than a property of product-related claims. Third, residual confounding remains after adjustment. The allegation group is a coarse proxy; the Public Use File lacks clinical specialty, procedure type, patient characteristics, and jurisdiction, and the structural overlap between the exposure and the adjustment variable further limits what the adjusted model can remove. Fourth, the file lacks claim narratives, device names, product identifiers, and individual practitioner identifiers, so the specific products involved cannot be characterized. Fifth, TOTALPMT is a banded practitioner-level payment represented by midpoints, and reports arising from the same underlying claim may each carry a separate value; findings were nevertheless similar among claims involving one practitioner. Sixth, the NPDB captures payments made for the benefit of practitioners and does not capture compensation involving only manufacturers, which may be precisely where device-centered claims resolve. Finally, because the unit of analysis is the report rather than the patient or the claim, counts and proportions describe reporting activity and cannot be read as patient-level incidence or severity, and the design cannot establish why the observed selection occurs.

## Conclusions

Physician malpractice payment reports carrying product or equipment codes were rare, accounting for approximately 1% of physician reports, and were coded with substantially lower injury severity and lower banded payments than other physician reports. The pattern persisted after adjustment for allegation group and in sensitivity analyses, but it describes the behavior of a coding system rather than the severity of product-associated patient harm.

We therefore recommend that NPDB product and equipment codes not be used as a standalone case definition for research or policy analysis of device- and product-related malpractice and that studies employing these fields state explicitly that they capture documented coding rather than product involvement. Work that aims to quantify compensation for product-associated injury will require linkage to claim narratives, device-event data, or court records, together with validation against a source that records device identity directly. The addition of more granular device and product fields in future public use releases would substantially improve the value of the NPDB for this question.

## Appendices

**Table 5:** NPDB Public Use File fields and codes used for classification NPDB, National Practitioner Data Bank; ALGNNATR, allegation group; TOTALPMT, NPDB Public Use File banded payment made by the payer for the named practitioner; ORIGYEAR, report origination years; LICNFELD, license field; OUTCOME, NPDB outcome categories; NUMBPRSN, number of practitioners; MD, Doctor of Medicine; DO, Doctor of Osteopathic Medicine

Construct	Field(s)	Values used
Study population	LICNFELD	10 (MD), 20 (DO)
Study period	ORIGYEAR	2019 through 2025 (sensitivity analysis: 2019 through 2023)
Product or equipment coding	ALGNNATR; ALEGATN1; ALEGATN2	ALGNNATR=80 (Equipment/Product Related), or ALEGATN1 or ALEGATN2=303, 320, 332, 704, 709, or 715
Intrinsic product subset	ALEGATN1; ALEGATN2	320, 704, or 715
Explicit product liability	ALEGATN1; ALEGATN2	715
Severe injury or death	OUTCOME	Significant permanent injury; major permanent injury; quadriplegia, brain damage, or lifelong care; death
Death	OUTCOME	Death
Payment	TOTALPMT	All released bands, each represented by its midpoint (highest, open-ended band assigned its lower bound)
Single-practitioner sensitivity analysis	NUMBPRSN	1
